# Gold Nanobeacons for Tracking Gene Silencing in Zebrafish

**DOI:** 10.3390/nano7010010

**Published:** 2017-01-11

**Authors:** Milton Cordeiro, Lara Carvalho, Joana Silva, Leonor Saúde, Alexandra R. Fernandes, Pedro V. Baptista

**Affiliations:** 1 UCIBIO, Departamento Ciências da Vida, Faculdade de Ciências e Tecnologia, Universidade Nova de Lisboa, 2829-516 Caparica, Portugal; m.cordeiro@campus.fct.unl.pt (M.C.); pvb0608@gmail.com (J.S.); ma.fernandes@fct.unl.pt (A.R.F.); 2 LAQV, REQUIMTE, Departamento de Química, Faculdade de Ciências e Tecnologia, Universidade Nova de Lisboa, 2829-516 Caparica, Portugal; 3 Instituto de Medicina Molecular e Instituto de Histologia e Biologia do Desenvolvimento, Faculdade de Medicina da Universidade de Lisboa, 1649-028 Lisboa, Portugal; lcarvalho@fm.ul.pt (L.C.); msaude@fm.ul.pt (L.S.)

**Keywords:** gold nanobeacons, gold nanoparticles, gene silencing, zebrafish, fli-EGFP

## Abstract

The use of gold nanoparticles for effective gene silencing has demonstrated its potential as a tool for gene expression experiments and for the treatment of several diseases. Here, we used a gold nanobeacon designed to specifically silence the enhanced green fluorescence protein (EGFP) mRNA in embryos of a fli-EGFP transgenic zebrafish line, while simultaneously allowing the tracking and localization of the silencing events via the beacon’s emission. Fluorescence imaging measurements demonstrated a decrease of the EGFP emission with a concomitant increase in the fluorescence of the Au-nanobeacon. Furthermore, microinjection of the Au-nanobeacon led to a negligible difference in mortality and malformations in comparison to the free oligonucleotide, indicating that this system is a biocompatible platform for the administration of gene silencing moieties. Together, these data illustrate the potential of Au-nanobeacons as tools for in vivo zebrafish gene modulation with low toxicity which may be used towards any gene of interest.

## 1. Introduction

The use of antisense oligonucleotides (ASO) as therapeutic agents was proposed for the first time by Zamecnik and Stephenson in 1978 [1]. These agents are used to specifically knockdown gene expression, through the hybridization and formation of a complex with a specific mRNA. The hybrid duplex (DNA-mRNA) is responsible for the recruitment of the RNAse H enzyme, which hydrolyzes the RNA portion of the duplex. The antisense DNA may then hybridize to another mRNA molecule [2], leading to gene expression knockdown. However, these molecules require the use of transfection agents to promote cell internalization, and upon entry, the activity of endogenous nucleases leads to short persistence of the antisense molecules (due to the antisense sequence hydrolysis). The use of gold nanoparticles (AuNPs) has shown the capacity to bypass these limitations [3]. Particularly, we have shown increased knockdown efficiency of gene expression (oligonucleotides have an added protection from enzymatic degradation), while simultaneously reducing the toxicity and immune response problems associated with the most common transfection systems. In addition, AuNPs have the capability to quench the fluorescence of nearby fluorophores in a distance-dependent manner. As such, the functionalization with fluorescence-labeled single-strand DNA (ssDNA) with a hairpin structure leads to fluorescence quenching due to the fluorophore’s proximity to the AuNP surface. Upon hybridization to the target sequence, the fluorophore breaks away from the surface of the AuNP, leading to a partial recovery of the fluorescence emission—gold nanobeacons (Au-nanobeacons) [4,5]. This dynamic formulation allows for the fluorescent detection of the silencing event, considering the partial fluorescence recovery upon hybridization to the target sequence. Additionally, Au-nanobeacons were demonstrated to be viable silencer agents both in in vitro and in vivo without toxicity [6]. However, further studies using more complex in vivo models are paramount to demonstrate the efficacy but also infer the induction of malformations/toxicity.

*Danio rerio* (zebrafish) is one of the most reliable and well-established in vivo models for a multitude of studies and screenings, including carcinogenesis and developmental studies, as well as toxicity assays [7,8,9,10], including environmental toxicity and nanoparticle toxicity [11,12]. The advantages of using this in vivo model are mainly their fast reproduction, the high number of fingerlings, the optical transparency of embryos which allows real-time monitoring of the development process and of fluorescence markers, the ease of manipulation and maintenance [7,13,14,15,16], and high homology to the human genome [17].

Here, we show that the Au-nanobeacons are suitable and efficient platforms for gene silencing in embryos of a fli-enhanced green fluorescence protein (fli-EGFP) transgenic zebrafish line [18,19], and constitute a valuable tool to track and localize in vivo as the silencing occurs without hampering the embryo’s development or killing the organism. This way, these Au-nanobeacons may become a valuable tool in zebrafish studies involving gene modulation.

## 2. Results and Discussion

### 2.1. Synthesis and Characterization of the Au-Nanoconjugates

**Figure 1:**
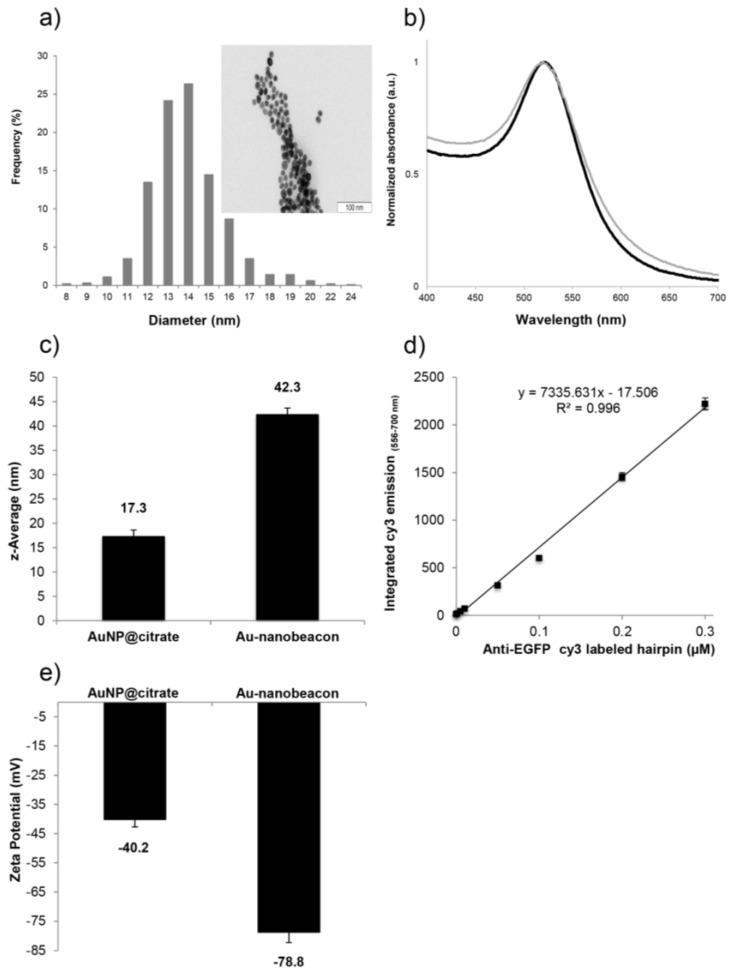
Characterization of the synthesized AuNP@citrate and Au-nanobeacon; (**a**) Size distribution of the synthesized AuNP@citrate, Inset: Transmission electron microscopy (TEM) image of the AuNP@citrate (scale bar: 100 nm); (**b**) Ultra-violet (UV)-Vis spectra of the AuNP@citrate (solid black line) and Au-nanobeacon (solid grey line); (**c**) Hydrodynamic diameter of AuNP@citrate and Au-nanobeacon; (**d**) Calibration curve for the quantification of the number of hairpins per PEGylated AuNPs; (**e**) Zeta potential of AuNP@citrate and Au-nanobeacon.

The Turkevich synthesis yielded citrate-capped AuNPs (AuNP@citrate) [20] with an average core size of 13.95 ± 1.79 nm (Figure 1a–c), which were functionalized with *O*-(2-Mercaptoethyl)-*O*′-methylhexa(ethylene glycol), C_15_H_32_O_7_S, 356.48 Da), generating PEGylated AuNPs (AuNP@PEG) [4,5]. A 30% surface coverage of the AuNP@citrate was used following the procedure described in [4], which allows for incorporation of the thiolated ssDNA molecules while conferring stability and increased biocompatibility. The AuNP@PEG was further functionalized with 6 ± 2 Cy3 labeled ssDNA oligonucleotides bearing the EGFP antisense sequence (see Figure 1d for calibration curve used), yielding Au-nanobeacons. The UV-visible (UV-Vis) absorption spectra showed an increase of the full width at half maximum of the localized surface plasmon resonance (LSPR) of the Au-nanobeacon compared to the AuNP@citrate (Figure 1b), indicating a broader size dispersion of AuNPs in solution. Dynamic light scattering (DLS) measurements (Figure 1c) showed an increase in the hydrodynamic diameter (expressed as the z-average) from 17.3 ± 0.6 nm for the AuNP@citrate to 42.3 ± 1.4 nm for the Au-nanobeacon due to the ssDNA stem-loop isotropic functionalization (on every side of the AuNPs) (Figure 1c,d), corroborated by the decrease in the zeta potential with a value of −78.8 ± 1.3 mV (Figure 1e). Together, these data show that the AuNPs were successfully functionalized and the Au-nanobeacon product is stable.

### 2.2. Silencing Efficiency

**Figure 2:**
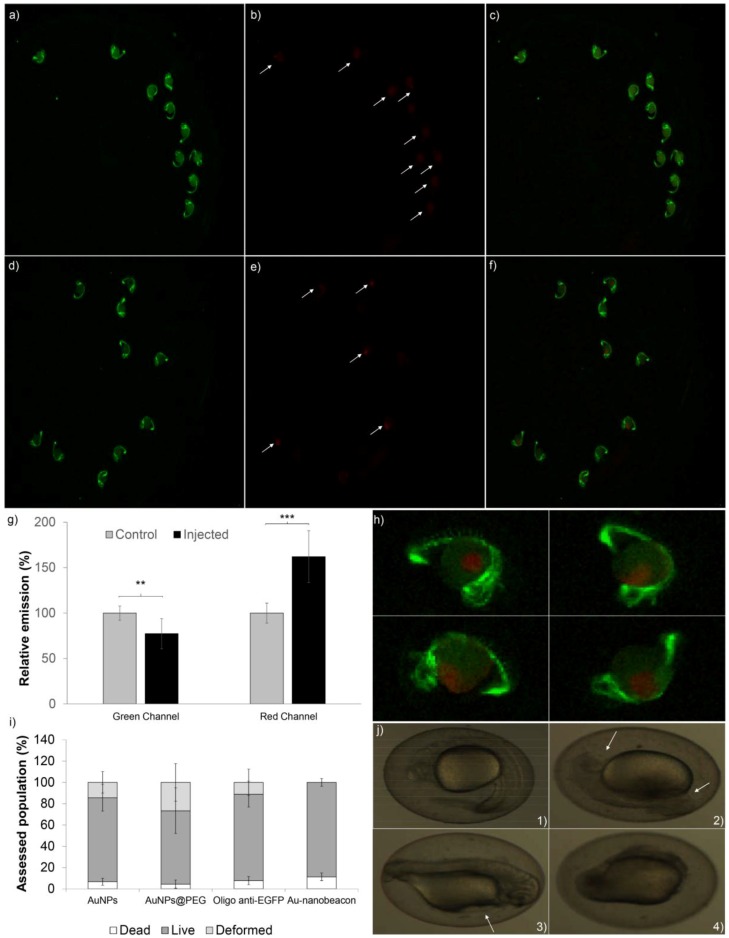
Au-nanobeacon silencing efficiency of the enhanced green fluorescence protein (EGFP) and acute toxicity assessment. Fluorescence imaging of whole embryos after injection (amplification 8.1×); (**a**) Green channel of control embryos; (**b**) Red channel of control embryos; (**c**) Merged channels for control embryos; (**d**) Green channel of injected embryos; (**e**) Red channel of injected embryos; (**f**) Merged channels for injected embryos (8.1× amplification); (**g**) Quantification of fluorescence in whole embryos using Image J. The results were normalized to the respective channel of the control. The data are expressed as mean ± standard deviation of five embryos (sample *t* test—*** for *p* < 0.05); (**h**) Zoom of 32.4× of the injected embryos’ merged channels (400% of 8.1×); (**i**) Quantification of death, survival and morphological malformations upon microinjection of AuNP@citrate, AuNP@PEG, Oligo anti-EGFP and Au-nanobeacon; Error bars corresponds to standard deviation of at least 50 embryos; (**j**) Example of embryos observed after microinjection of AuNP@citrate, AuNP@PEG, Oligo anti-EGFP and Au-nanobeacon: (**j1**) Normal embryo; (**j2**) Head and tail malformation; (**j3**) Pericardial edema; (**j4**) Underdeveloped embryo.

The silencing efficiency of the synthesized Au-nanobeacon was evaluated in an in vivo model, the fli-EGFP transgenic zebrafish. The level of silencing was correlated with the EGFP emission (green channel) and the Au-nanobeacon output was correlated with the Cy3 emissions (red channel). The injection of 150 nM of Au-nanobeacon led to a 22.7% ± 16.6% reduction of the emissions in the green channel (Figure 2a vs. Figure 2d), with a concomitant increase of 62.2% ± 28.0% in the red channel in comparison to the control (Figure 2b vs. Figure 2e)—the white arrows highlight the measured embryos). The overlay of the green and red channels of the injected group allows the co-localization of both the green and red channels—Figure 2c, whereas the overlay of both channels in the control group only allows for the observation of the green channel—Figure 2f. See Figure 2h for a amplification of affected zebrafish. The intensity quantification is represented in Figure 2g). These results suggest that injection of the designed Au-nanobeacon causes a decrease in the green channel intensity, which correlates with the hybridization of the Au-nanobeacon to the target mRNA, leading to a reduction of the gene expression. The red channel shows a higher intensity in the injected group, suggesting that target recognition induces the opening of the hairpin, resulting in the separation of the Cy3 and the AuNP (quencher). This allows the fluorophore to partially recover its emission and to monitor the intracellular interactions of AuNPs with the target and the specific silencing of gene expression, as previously demonstrated in tumor cell lines [5,6,7] as well as in more complex models, such as hydra and mice [6,21,22,23].

The silencing efficiency of the Au-nanobeacon was compared with the microinjection of the free antisense oligonucleotide anti-EGFP at the same concentration administered through the AuNPs. For the free EGFP antisense oligonucleotide, the reduction of the EGFP emission was negligible at 2.8% ± 2.3% (data not shown), in comparison to the 22.7% ± 16.6% obtained with the Au-nanobeacon (Figure 2g), demonstrating the advantage of the Au-nanobeacon for anti-sense oligonucleotide delivery when compared to the administration of the free oligonucleotide.

### 2.3. Toxicity in Zebrafish Embryos

Acute toxicity is of paramount importance for the successful application of the developed Au-nanobeacon for gene therapy. Toxicity was assessed based on the determination of the percentage of survival, death and malformation 24 h post-fertilization in the presence of the different nanoconjugates (at least 50 embryos per assay). Results presented in Figure 2i shows that the injection of the Au-nanobeacon led to a mortality of 11.3% ± 3.6%, which is the highest among the different conditions tested, in agreement with previously published work [24]. Administration of the same concentration of free oligonucleotide led to 84.1% ± 12.2% survival (Figure 2i). Furthermore, malformations were detected in 10% ± 12.5% after the administration of free oligonucleotide. Injection of free oligonucleotide resulted in: Normal development (Figure 2j1), head and tail malformations (Figure 2j2), pericardial edema (Figure 2j3) as well as totally undeveloped embryos (Figure 2j4). These findings demonstrate an interference in the embryonic development process of zebrafish and greater toxicity associated with the administration of such free molecules. It has been demonstrated that AuNPs with different coatings appear to have no toxic effects in zebrafish embryos and concluded that this absence of toxicity was due to the lack of internalization of the AuNPs [24,25]. However, in our study, upon the injection of the Au-nanoconjugates directly into the zebrafish embryos, we demonstrated the in vivo silencing potential (loss of green fluorescence simultaneously with the increase of red fluorescence due to the opening of the hairpin) with relatively low toxicity/malformations.

## 3. Materials and Methods

### 3.1. Synthesis and Functionalization of AuNPs

AuNPs were synthesized using the procedure described by Turkevich and later optimized by Lee and Meisel [20,26], yielding citrate capped AuNP (AuNP@citrate). The AuNP@citrate were functionalized as described in [4,5]. Briefly, AuNPs were functionalized with a 30% surface coverage using *O*-(2-Mercaptoethyl)-*O′*-methyl-hexa(ethylene glycol) and further functionalized with a 3′ Cy3 fluorophore labeled hairpin-ssDNA (5′-tttgccgctcctggacgtagccttcgggggcaaa-3′) bearing a thiol on the 5′ end, as to act as an Au-nanobeacon. Direct excitation of Cy3 at 550 nm was used to quantify the unbound ssDNA in the supernatants collected during the washing steps. The emission spectra of the supernatants collected from the washing steps was converted into molar concentration through the interpolation in a calibration curve, prepared with known concentrations of oligonucleotide in the same reactional conditions as the supernatants. The difference between the added oligonucleotides and the washed oligonucleotide was used to calculate the average number of functionalized oligonucleotides. The Au-nanobeacons were characterized by UV-Vis absorption spectroscopy, zeta potential and by DLS as previously described [5]. The zeta potential and DLS assays were performed in milli-Q water.

### 3.2. Ethics Statement

Experiments involving animals were approved by the Animal User and Ethical Committees at Instituto de Medicina Molecular (Lisboa, Portugal), according with directives from Direcção Geral Veterinária.

### 3.3. Zebrafish Line

The fli-EGFP transgenic zebrafish [18,19] line was used as animal model and maintained in a re-circulating system with a 14 h/day and 10 h/night cycle at 28 °C. After fertilization, the embryos were collected as described in the Zebrafish Book [19] and maintained in E3 zebrafish embryo medium (5.03 mM NaCl, 0.17 mM KCl, 0.33 mM CaCl_2_·H_2_O, 0.33 mM MgSO_4_·7H_2_O, 0.1% (*w*/*v*) methylene blue) at 28 °C, until reaching the developmental stage required.

### 3.4. Microinjection of Zebrafish Embryos

The fli-EGFP transgenic zebrafish line was chosen due to its EGFP stained vasculature, providing an easy model to evaluate the silencing efficacy of the Au-nanobeacon. Post-fertilized fli-EGFP embryos were collect and embryos at the sphere stage were divided in different groups and injected with: 50 nM of AuNPs@citrate and 50 nM of AuNPs@PEG as controls and 150 nM of Au-nanobeacon, using a PV-820 Pico-injector (World Precision Instruments, Sarasota, FL, USA) and a Narashige micromanipulator. The free oligonucleotides molecules were also injected at the same concentration administered in the Au-nanobeacon. The embryos were incubated in 90 cm Petri dishes (Sarstedt, Germany) at 28 °C using embryo medium, until 24 h post-fertilization, a time-point where blood vessels are already formed and therefore the levels of EGFP expression could be evaluated.

### 3.5. Silencing and Imaging

Live embryos were anesthetized by addition of 500 µL of 1% (*v*/*v*) Tricaine (Western Chemical, Ferndale, WA, USA) per 25 mL of embryo medium in 90 cm Petri dishes. Pictures were taken using an Olympus MVX10 magnifying glass with an incorporated AxioCam ICc3 (Olympus, Tokyo, Japan) and acquired by ZEN software, Blue Edition 2011 (Oberkochen, Germany). EGFP and Cy3 emission was determined by the quantification of pixels’ intensity using the software ImageJ 1.49v (Bethesda, MD, USA). Each experiment was normalized to the respective control.

### 3.6. Toxicity Assessment in Zebrafish Embryos

To evaluate the toxicity effects of the tested constructs, the mortality/survival rates and the number of morphological malformations in zebrafish embryos were assessed after microinjection. AuNPs@citrate, AuNPs@PEG, Au-nanobeacon and free oligonucleotide were injected in the same conditions as described previously, and effects evaluated 24 h post-fertilization. The number of malformations, mortality and survival percentages were determined in comparison to injection of 10 mM phosphate buffer pH 8.

## 4. Conclusions

In this study, we successfully applied gold nanoparticles functionalized with an ssDNA with a hairpin conformation bearing the Cy3 fluorophore (Au-nanobeacon), which act as antisense oligonucleotides towards endogenous EGFP mRNA. We observed a reduction of the EGFP emission, indicating a downregulation of EGFP expression, with a concomitant increase of the fluorophore emission. No changes in the expression of EGFP were seen when the free oligonucleotide molecules were injected, indicating that the AuNPs are a suitable vehicle for antisense oligonucleotides. We demonstrate that the Au-nanobeacon shows eight times higher silencing capability in comparison to the free oligonucleotide, and is more biocompatible than AuNP@citrate and AuNP@PEG. This is the first proof-of-concept of using Au-nanobeacons for gene silencing in an in vivo zebrafish model.
